# Enhanced In Vivo Wound Healing Efficacy of a Novel Piperine-Containing Bioactive Hydrogel in Excision Wound Rat Model

**DOI:** 10.3390/molecules28020545

**Published:** 2023-01-05

**Authors:** Saeed Ali Alsareii, Javed Ahmad, Ahmad Umar, Mohammad Zaki Ahmad, Ibrahim Ahmed Shaikh

**Affiliations:** 1 Department of Surgery, College of Medicine, Najran University, Najran 11001, Saudi Arabia; 2 Department of Pharmaceutics, College of Pharmacy, Najran University, Najran 11001, Saudi Arabia; 3 Department of Chemistry, Faculty of Science and Arts, Promising Centre for Sensors and Electronic Devices, Najran University, Najran 11001, Saudi Arabia; 4 Department of Pharmacology, College of Pharmacy, Najran University, Najran 11001, Saudi Arabia

**Keywords:** piperine, hydrogel, carbopol 934, *Aloe vera* gel, wound healing, excision wound

## Abstract

These days an extensive amount of the attention of researchers is focused towards exploring bioactive compounds of natural or herbal origin for therapeutic intervention in different ailments of significant importance. One such novel bioactive compound that has a variety of biological properties, including anti-inflammatory and antioxidant activities, is piperine. However, until today, piperine has not been explored for its potential to improve inflammation and enhance healing in acute and chronic wounds. Therefore, the present study aimed to investigate the wound healing potential of piperine hydrogel formulation after topical application. Hydrogels fit the need for a depot system at the wound bed, where they ensure a consistent supply of therapeutic agents enclosed in their cross-linked network matrices. In the present study, piperine-containing carbopol 934 hydrogels mixed with *Aloe vera* gels of different gel strengths were prepared and characterized for rheological behavior, spreadability, extrudability, and percent (%) content uniformity. Furthermore, the wound healing potential of the developed formulation system was explored utilizing the excision wound healing model. The results of an in vivo study and histopathological examination revealed early and intrinsic healing of wounds with the piperine-containing bioactive hydrogel system compared to the bioactive hydrogel system without piperine. Therefore, the study’s findings establish that the piperine-containing bioactive hydrogel system is a promising therapeutic approach for wound healing application that should be diligently considered for clinical transferability.

## 1. Introduction

The occurrence of a wound seems to be a normal incident in every person’s day-to-day life. However, its healing has to deal with a cascade of comprehensive pathophysiological processes [1]. Wound healing is predominantly perceived as a normal biological process in the human body, which is accomplished through four highly sequenced and time-destined precisely programmed phases, including hemostasis, inflammation, proliferation, and remodeling [1,2,3]. Therefore, for successful wound healing, all these phases are supposed to occur in a definite sequence and period. Acute wound healing usually happens effectively in a healthy individual without any need for drug delivery aid at any stage of the pathological process [3,4]. Nonetheless, this course of wound healing is susceptible to many obstacles that hamper the process of wound repair, which include the condensed ability to re-establish a blood supply to the injury site, reduced oxygen supply, highly reactive oxygen species, high levels of pro-inflammatory cytokines, bacterial infection, and high pH [2,3,4]. All these factors ultimately lead to wound chronicity, and treating such wounds is challenging. Indeed, human skin wounds have become a threat to global public health and the economy [5,6]. In the United States alone, approximately 6.5 million patients are affected by chronic wounds. It has been estimated that more than USD 25 billion is spent annually on treating chronic wounds, which is an alarming figure [5,6].

The mechanism of wound healing therapy is usually aimed at restoring the dysregulated signaling processes, boosting re-epithelialization, diminishing inflammation, improving oxygen levels, reducing oxidative stress, stimulating angiogenesis and fibroblast migration along with the concomitant impedance of microbial invasion in the wound milieu [3,4,7,8]. To accomplish these targets, consistent and sustained release of the therapeutic agent is required at the wound bed from a depot-like system. Hydrogels perfectly fit this need for a depot system where they ensure a consistent supply of therapeutic agents enclosed in their crosslinked network matrices. Hydrogels are crosslinked three-dimensional network structures that can remarkably imbibe large quantities of water [9,10]. Their unique porous structure facilitates gaseous exchange and the maintenance of fluid balance in the wound area. Moreover, because of their permeable crosslinked matrices, essential functionality, including cell migration, growth, and maturation, is viable at the wound site, which could help to a large extent in wound healing [11]. The physicochemical properties of the polymers govern the characteristic properties and functionality of the hydrogel they are made up of. Depending on the source, these polymers can be naturally derived or synthetic. Preceding studies have demonstrated that wounds treated with Cx43 AsODN Pluronic gels were less inflamed, produced fewer exudates, and had enhanced re-epithelization and granulation tissue formation [12,13]. In another study, doxycycline was delivered via a disulfide crosslinked PEG, and the prepared hydrogel delivery system confirmed a sustained release of doxycycline for up to 10 days along with enhanced skin permeation of doxycycline that substantially promoted the wound healing process [14]. Nonetheless, hydrogels offer an edge over other carriers for topical application by creating a prerequisite moist environment in the wound area and allowing the controlled and sustained release of a therapeutic agent over a long duration of time. Therefore, in the present study, hydrogels were selected as a delivery system for investigating the therapeutic activity of a novel bioactive compound in the excision wound healing model.

Recently, there has been an increasing interest in exploring the potential of compounds of natural or herbal origin in improving inflammation and enhancing healing in acute and chronic wounds. Curcumin is one such successfully clinically exploited molecule of herbal origin [15]. One novel bioactive compound with various biological activities, notably anti-inflammatory and antioxidant, is piperine [16,17]. It is the primary plant alkaloid obtained from black pepper *(Piper nigrum*) and long pepper (*Piper longum*) [16]. Piperine is reported to increase TGF-β level, facilitate collagen repair, and prevent NF-ĸB activation in rodent periodontal tissues [18]. However, to date, it has not been explored for its wound healing potential; therefore, this study aimed to investigate the wound healing potential of topical piperine hydrogel formulation. In the present study, piperine-containing carbopol 934 hydrogels mixed with *Aloe vera* gel of different gel strengths was prepared and characterized for rheological behavior, spreadability, extrudability, and % content uniformity. The wound healing potential of the developed formulation system was explored for the first time in the current investigation through excision wounds in Wistar rats.

## 2. Results and Discussion

### 2.1. Preparation of Carbopol Hydrogel and Gel Strength Determination

The characteristic properties of hydrogels of substantial significance in treating wounds are gel strength, spreadability, and thixotropic characteristics. Ideally, the hydrogel strength should lie in a range of 25–50 s for topical administration because if it is less than 25 s, then the gel would not be able to adhere appropriately to the wound, and if it is more than 50 s, then it will be difficult to spread the gel uniformly over the wound bed. In the present study, carbopol hydrogel of different strengths was prepared: 0.2% *w*/*w*, 0.3% *w*/*w*, and 0.4% *w*/*w*, and the gel strength was found to be 38.94 ± 1.17 s, 41.05 ± 1.03 s, and 43.31 ± 1.26 s respectively. The carbopol 934 hydrogels of gel strength of 0.3% *w*/*w* were selected because their optimal characteristic is desirable for topical hydrogel preparation, mainly exploited in wound healing applications [19,20].

### 2.2. Preparation and Characterization of Piperine-Containing Bioactive Hydrogel

**Figure 1:**
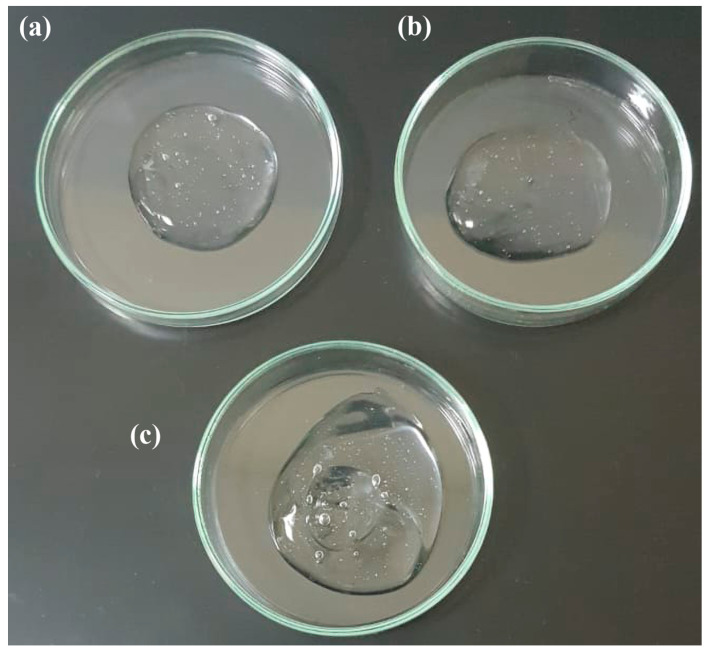
Illustration shows different types of prepared hydrogel system. (**a**) Carbopol 934 hydrogel. (**b**) Placebo bioactive hydrogel (excluding piperine). (**c**) Piperine-containing bioactive hydrogel.

The piperine-containing bioactive hydrogel system was prepared as described in Section 3.2. The % composition of the optimized formulation system for wound healing application is shown in Table 1 and different types of gel samples are shown in Figure 1.

**Figure 2:**
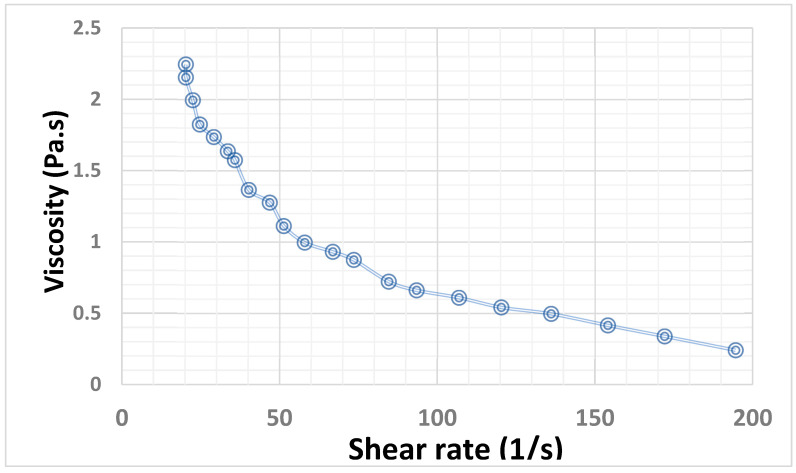
Rheology of piperine-containing bioactive hydrogel system.

The developed piperine-containing bioactive hydrogel system was characterized for its rheology profile. The unique attribute of hydrogels is their sol–gel inter-convertible nature or thixotropic property. Due to this, they convert into a softer and spreadable sol form on the application of shear rate/stress, and once that force is withdrawn, they adhere to the surface over which they were spread in the same way as a stiff gel. This rheological behavior (Figure 1) of the developed formulation is helpful in imparting the spreadability and extrudability characteristics to the piperine-containing bioactive hydrogel system. To evaluate the rheological behavior of the developed piperine-containing bioactive hydrogel system, the rotational viscometer of a parallel plate was used as described in Section 3.3. Figure 2 illustrates the rheological behavior of the prepared piperine-containing bioactive hydrogel system, which can be seen undergoing gel-to-sol transformation and exhibiting a prominent shear thinning under the influence of shear stress. Gradual recovery to the gel state on the withdrawal of stress was considerably noticed during the rheology study of the hydrogel system. It was observed that the hydrogel system has a thixotropic characteristic, non-Newtonian and pseudoplastic behavior that is greatly desirable for ideal topical formulation [19,20,21,22].

The prepared piperine-containing bioactive hydrogel system was sufficiently viscous to be easily able to adhere to the wound bed. As well as adherence, a great difficulty encountered while applying a topical preparation is that it is painful to apply or rub the gel over the wound area; therefore, the hydrogel is supposed to have great spreadability so that the minimum possible manual force is required to spread it over the wound. Easy spreadability of the hydrogel formulation is highly desirable. To exhibit good spreadability, pseudoplastic rheological behavior is desirable for the topical formulation, particularly for wound healing applications. The developed piperine-containing bioactive hydrogel system showed pseudoplastic behavior as seen in Figure 1. The pseudoplastic behavior of the developed formulation is helpful to attain the desired spreadability [20,23]. The spreadability factor of the developed piperine-containing bioactive hydrogel system was found to be 0.56 ± 0.02 cm^2^/g, which seems to be optimum for easy spreading of the gel over the wound area. The great ease of exuding piperine-containing bioactive hydrogel system from a collapsible tube demonstrated the appreciable extrudability characteristic of the developed formulation system for wound healing application.

Furthermore, the % drug content and content uniformity of the hydrogel system were calculated. It was observed that piperine has uniform dispersion (99.97 ± 0.23%) in the developed formulation system. The % content of piperine in the developed formulation system was found to be 99.87 ± 0.41%.

### 2.3. Wound Healing Activity

**Figure 3:**
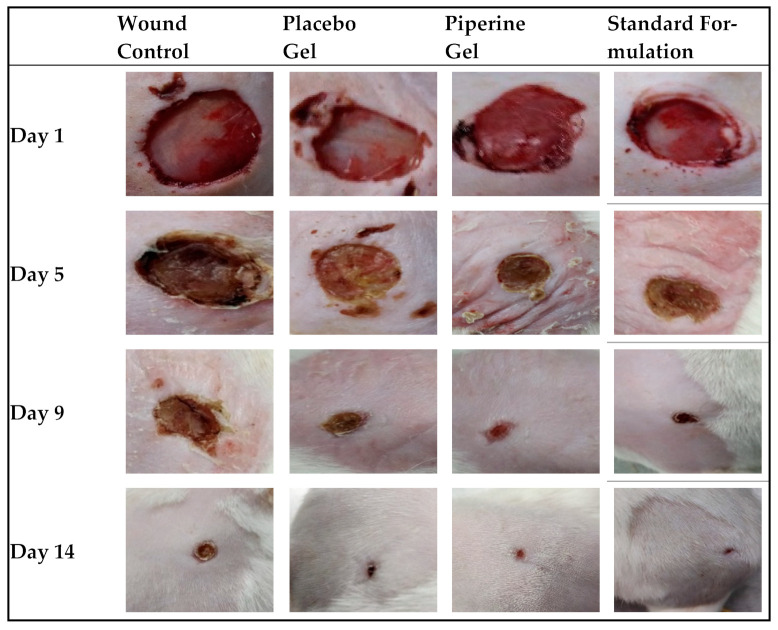
Qualitative trend of the wound healing process of rat groups receiving no treatment (control), placebo gel, piperine gel, and standard marketed formulation. The photos were taken on Day 1, Day 5, Day 9, and Day 14 of treatment.

Recent years have seen an increase in research into the wound healing potential of natural products with anti-inflammatory, antioxidant, antibacterial, and pro-collagen synthesis actions. Phytochemicals can speed up the recovery time from wounds because of their medicinal properties [24,25]. The wound healing potential of piperine was explored for the first time in the current investigation. As described in Section 3.4, after induction of the wounds, animals were divided into the following four groups containing six animals in each group, group I represents the negative control (wound-induced rodents) with no treatment; in group II the rats were treated with placebo bioactive gel containing *Aloe vera* juice (excluding piperine); in group III, the rats were treated with standard marketed formulation (silver sulfadiazine cream of strength 1% *w*/*w*); and rats in group IV were treated with the piperine-containing bioactive hydrogel system (0.5% *w*/*w* of piperine). Furthermore, as *Aloe vera* juice is also established as aiding in wound healing, a placebo aloe vera–carbopol 934 hydrogel system (group II) was tested to assess its wound healing potential in the absence of piperine. The results of the wound healing activity are shown in Figure 3. It demonstrates the in vivo wound healing effect of the topically applied formulations (groups II, III, and IV).

**Figure 4:**
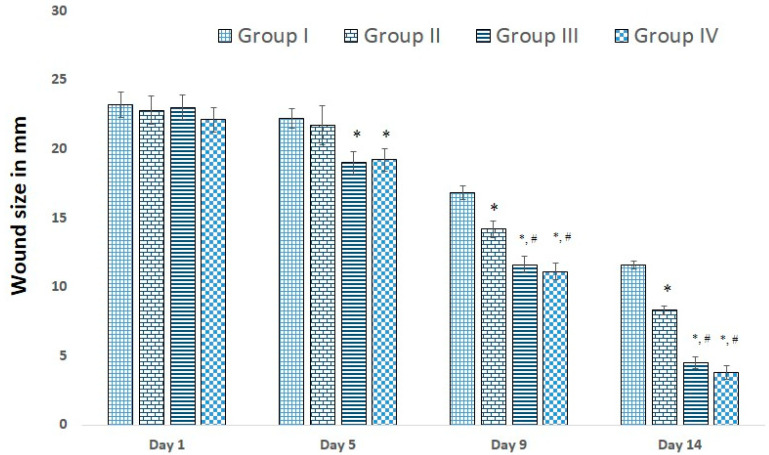
Illustrating the progressive contraction of wound size in different groups. * *p* < 0.01 vs. control (Group I), ^#^ *p* < 0.01 vs. placebo (Group II).

The wound contraction area was examined at different time intervals, i.e., 1st, 5th, 9th, and 14th days of wound induction. As we had anticipated, the wound area was not significantly (*p* > 0.05) different between all the groups (~23.32 ± 1 mm) on day 1. To evaluate the process of wound healing and compare their capacities to improve the rate of wound healing, measurement of the wound sizes and % wound healing was performed on different days of the treatment. The qualitative trend of wound healing in treated rat groups is demonstrated in Figure 4.

As is clear, the process of wound healing was typically initiated and progressed in all groups. However, the rate of wound healing was significant (*p* < 0.01) in all treatment groups (group II, III, and IV) compared to the negative control (group I, no treatment). The wound size was around ~23.32 ± 1.00 mm on the first day (Day 1) in all the groups, which confirms no wound healing (0%) percentage. The trend of wound sizes decreased, and wound healing percentages increased in all treatment groups (groups II, III, and IV). Wound sizes decreased, and wound healing percentages increased significantly (*p* < 0.01) from Day 5 to Day 14 in groups III and IV compared to group I (Figure 3). From Day 5 till the end of the treatment (Day 14), all three treatments (group II, group III, and group IV) showed reduced wound sizes and better healing percentages in comparison to the negative control (group I) and placebo treatment group (group II). The wound sizes were the smallest (Figure 1), and the wound healing percentages were the maximum on Day 14 of treatment in the groups receiving the standard marketed formulation (group IV), followed by the developed piperine-containing bioactive hydrogel (group III) and placebo gel (group II). However, the wound size in the developed piperine-containing bioactive gel group was almost accomplished by Day 14 (wound sizes were 4.5 ± 0.5 mm), which was comparable to the standard marketed preparation (3.8 ± 0.4 mm). The percentage of wound contraction in different treatments is shown in Table 2.

The results show that on the 14th day, approximately 50% more than 80% of wound contraction was seen in animals treated with piperine-containing bioactive gel and standard marketed preparation on Day 9 and Day 14, respectively.

### 2.4. Histopathology

**Figure 5:**
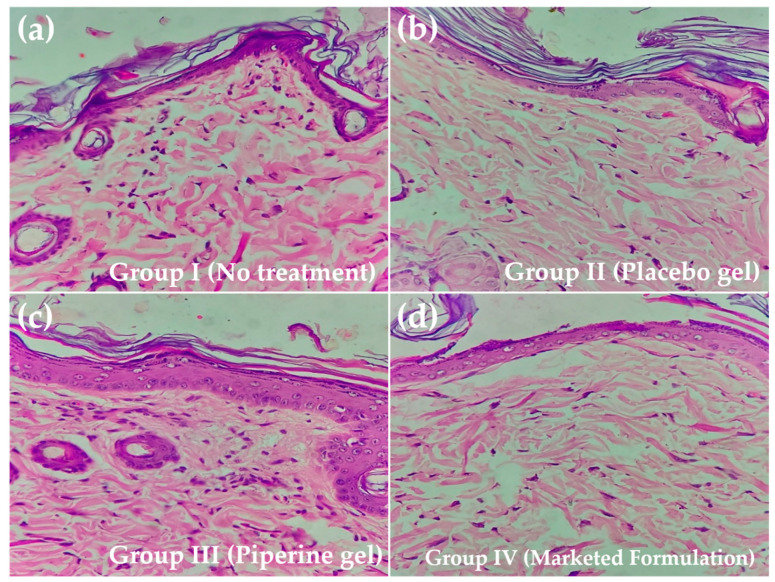
Histopathology analysis of newly healed tissue at day 14, tissues stained with hematoxylin–eosin: (**a**) superficial dermis shows inflammatory cell infiltration, (**b**) normal epidermis and dermis shows normally arranged fibro-connective tissue with interspersed skin adnexal structures and pigmentation, (**c**,**d**) section shows focal acanthosis in epidermis lined by keratinized stratified squamous epithelium.

The results of the histopathological evaluation of skin samples treated with different formulations (including skin samples of the no treatment group) on day 14 post-wounding are shown in Figure 5. The results are evidence of marked granulation and diminished inflammation in the positive control. The samples of the group treated with the placebo and standard marketed preparation showed signs of collagen and re-epithelization, but these findings were more pronounced in samples treated with the piperine-containing bioactive hydrogel system. Figure 4 confirms the piperine-containing hydrogel system’s substantial wound healing potential with widespread collagen fibre [24] and the formation of the thick epidermal layer and papillary dermis [24,25,26]. The significant healing of wounds with the piperine-containing hydrogel system is attributed to the augmented and controlled accessibility of piperine to the wound bed utilizing the hydrogel formulation that hastens the process of the formation of an organized, cellular structure in newly healed skin tissues.

**Figure 6:**
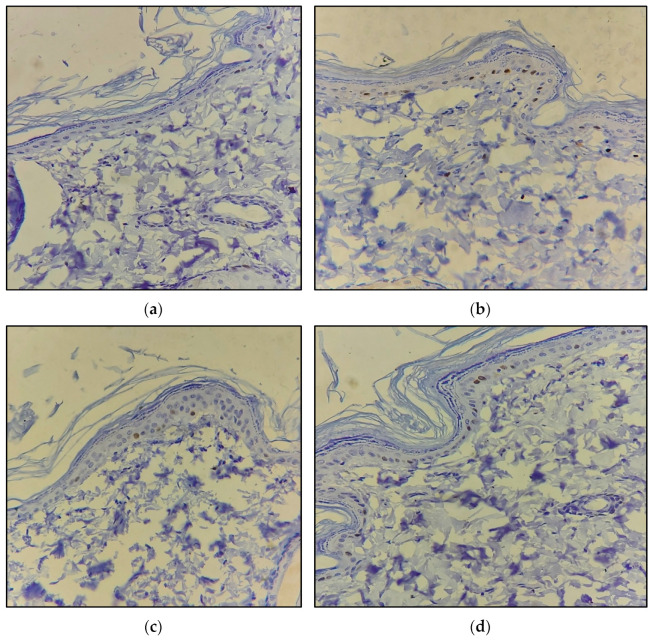
Section examined for IHC expression of Ki67 shows (**a**) wound control: negative expression of Ki67 in epidermis and dermis; (**b**) placebo bioactive gel: focal nuclear positive expression of Ki67 in epidermis basal layer and negative expression in dermis; (**c**) piperine-containing bioactive gel: focal positive expression of IHC Ki67 in epidermis basal layer and negative expression in dermis; (**d**) standard marketed formulation: focal positive expression of IHC Ki67 in epidermis basal layer and negative expression in dermis; (IHC Ki67, 400X).

The expression of cell proliferation-associated nuclear antigen Ki67 was evaluated using immunohistochemistry to identify the proliferating keratinocytes. The results showed that there was negative expression of Ki67 in the wound control group. Furthermore, the placebo bioactive gel group containing aloe vera showed the focal nuclear expression of Ki67 in the epidermal basal layer indicating active localized proliferation. The group treated with the piperine-containing bioactive gel exhibited the focal positive expression of Ki67 in the epidermal basal layer indicating active localized proliferation induced by the piperine gel. Similarly, positive and relative higher Ki67 expression was also observed in the basal layer of the skin tissue of animals treated with the standard marketed formulation. The Ki67 expression has been used as an indicator of the effectiveness of the repair process [27]. Therefore, the results of the analysis indicate the positive effect of piperine gel on the tissue repair process at the wound site as compared to the control and placebo groups (Figure 6).

**Figure 7:**
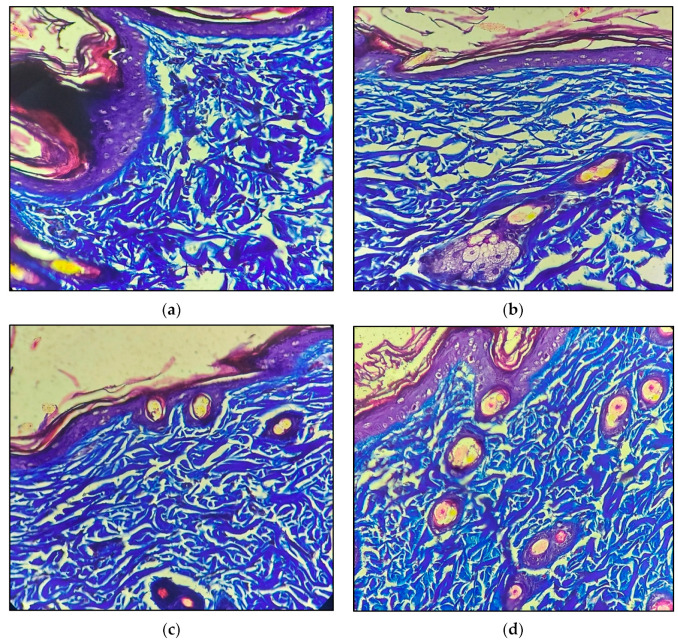
Result of MT staining in skin tissue of different treatment groups; (**a**) wound control: mildly haphazardly, loosely arranged collagen bundles in the dermis; (**b**) placebo bioactive gel: loosely arranged thin collagen bundles in the dermis; (**c**) piperine-containing bioactive gel: normally arranged and thick collagen bundle in dermis; (**d**) standard marketed formulation: normally arranged and thick collagen bundle in dermis (MT, 400X).

Collagen is an important component of the extracellular matrix that plays a significant role in the wound healing process [28,29,30,31,32]. The collagen deposition in the healing tissue was analysed using MT staining. The results showed that the tissue treated with the aloe-vera-juice-containing placebo bioactive gel group had a loosely arranged thin collagen bundle in the dermin, whereas there was a thick collagen bundle present in an arranged fashion in the tissues treated with the piperine-containing bioactive gel and standard marketed formulation. The results indicated that the two treatments promoted wound healing by facilitating collagen deposition (Figure 7).

An animal model reflects its ability when it replicates normal human processes and diseases, and it is called a true success of an animal model. The therapeutic efficacy of bioactive agents in rodent models of wound healing has been difficult to study because the contraction of the wound is rapid in rodents, leading to the rapid closure of defects [24,25,26,27,28,29,30,31,32]. This comparative study was designed to demonstrate the internal and epidermal healing potential of a piperine-containing hydrogel and its conventional formulation compared to no drug treatment. The study showed that skin wounds in rats allow healing through the processes of granulation and epithelialization while maximizing the effects of contraction. The wounds in the negative control rats took a prolonged time to complete wound closure and lesser collagen deposition compared to the treated animal groups. The animals treated with the piperine-containing hydrogel had significantly enhanced wound healing effects on rat models and helped in the contraction of the wound area from day 1 to 14 compared to the untreated rat group. The complete epithelization period was significantly smaller in treated groups compared to the untreated group, as evident from wound size, % wound contraction, and histopathological and immunohistochemical studies. It was observed that the piperine-containing hydrogel showed a comparable wound healing effect with respect to the standard marketed formulation.

## 3. Materials and Method

### 3.1. Materials

Piperine, triethanolamine, and glycerol were obtained from Sigma Aldrich (Taufkirchen, Germany). Carbopol 934 was obtained from Lubrizol (Wickliffe, OH, USA). Other ingredients and chemicals utilized in this investigation were of pharmaceutical and analytical grade.

### 3.2. Preparation of Carbopol Hydrogel and Gel Strength Determination

Carbopol 934 was used as a gelling agent for preparing a topical hydrogel system. Carbopol 934 hydrogel was prepared at different concentrations (0.2% *w*/*w*, 0.3% *w*/*w*, and 0.40% *w*/*w*) by dissolving 0.2 g, 0.3 g, and 0.4 g of it in 90 g of double distilled water and then making the final weight up to 100 g. These mixtures were kept overnight to achieve complete swelling and homogeneity of carbopol 934 into an aqueous system [33,34]. Triethanolamine was added drop-by-drop into an aqueous dispersion system of carbopol 934 to convert it into a hydrogel system. The prepared carbopol 934 hydrogel was evaluated for gel strength determination [35,36]. Briefly, carbopol 934 hydrogel weighing 60 g was placed in a 100 mL graduated measuring cylinder (1.5 inches in diameter). Subsequently, a disc (having a diameter of 3 cm and thickness of 3 mm) was placed on the gel surface with 30 g of weight mounted on it. Gel strength was determined by the time the disc took (in seconds) to sink 5 cm into the gel from the surface.

### 3.3. Preparation of Piperine-Containing Bioactive Hydrogel

An accurately weighed amount of carbopol 934 was uniformly dispersed into double distilled water with consistent stirring and homogenization. Subsequently, triethanolamine (2–3 drops) was poured into the mixture to neutralize the aqueous dispersion system of carbopol 934 into a hydrogel system. An accurately weighed amount of piperine was dissolved in the required quantity of glycerine and uniformly mixed into a hydrogel system of carbopol 934 to provide a humectant effect. Finally, an accurately weighed amount of *Aloe vera* juice was added further to divulge an appreciated emollient and anti-inflammatory effect to the prepared piperine-containing bioactive hydrogel system [33,34].

### 3.4. Characterization of Piperine-Containing Bioactive Hydrogel

The prepared piperine-containing hydrogel bioactive hydrogel system was evaluated for different physicochemical characteristics, including viscosity, rheology, spreadability, extrudability, and % content uniformity [33,34].

#### 3.4.1. Viscosity

The piperine-containing bioactive hydrogel system was tested for viscosity using a Brookfield viscometer (S-62, model LVDV-E) at 25 °C with the spindle speed of the viscometer rotated at 12 rpm [33,34].

#### 3.4.2. Rheology

A rotational viscometer with a parallel plate (BohlinVisco 88, Malvern Instruments Ltd., Malvern, UK) was employed to estimate a rheological profile of the prepared piperine-containing bioactive hydrogel system at room temperature. The thixotropic characteristic of the developed piperine-containing bioactive hydrogel system was studied using software (BohlinR6.51.0.3) [33,34].

#### 3.4.3. Spreadability

To evaluate the spreadability of piperine-containing bioactive hydrogel system, 0.5 g of it was placed between two already weighted concentric glass plates. Consequently, weight was added to the upper glass plate at an interval of 60 s. The spreading diameter of piperine-containing bioactive hydrogel system was noted after each weight addition. The spreadability factor was calculated by plotting the graph between the spreading areas versus the applied weight [36].

#### 3.4.4. Extrudability

For assessing the extrudability characteristics of piperine-containing bioactive hydrogel system, it was filled in a sealed collapsible tube and forced diligently at the crimped end. After the cap’s opening, the gel extrudes out in small proportion until the applied force is restrained. The force required to extrude out this small sample over a specific time was calculated to assess the extrudability efficiency of the prepared piperine-containing bioactive hydrogel system.

#### 3.4.5. Analysis of % Content of Piperine and Content Uniformity of Piperine in Hydrogel

The piperine-containing bioactive hydrogel system weighing 0.5 g was collected from three distinct regions of the developed topical formulation for the healing activity in an excisional wound. Each of these collected samples was dissolved in 10 mL of methanol for half an hour, followed by centrifugation of this extract at 3000 rpm for 15 min [36]. The supernatant was filtered, and the piperine was checked for content uniformity by quantifying the sample through UV spectrophotometric analysis at λmax 342 nm. The experiment was performed in triplicate.

### 3.5. In Vivo Study: Wound Healing Activity

#### 3.5.1. Animals

The wound healing potential of prepared piperine-containing bioactive hydrogel system was investigated in excision wounds in an animal model [37]. The approval of animal protocol (approval no: 443-42-59216-DS) for wound healing studies was taken from the institutional animal ethical committee (Najran University, Najran, Saudi Arabia) and their guidelines were followed throughout the studies. Albino Wistar rats (6–8 weeks, weighing 200–250 g) were used to assess the wound healing activity of the developed piperine-containing bioactive hydrogel system. The number of animals per group were calculated using G*Power, statistical power calculator tool. The animals had free access to a laboratory diet and water ad libitum. The animals were stored in a laboratory maintained at standard temperature and humidity conditions (temperature: 25 ± 2 °C, 55 ± 5% RH).

#### 3.5.2. Excision Wound Model

Inducing wounds in an animal model, the animals were anaesthetized using a mixture of ketamine and xylazine (80 mg/kg and 10 mg/kg, respectively). The anaesthetized animals were shaved to remove the hair. Their skin layer was cut, and an excision wound of 23 ± 1 mm^2^ area with an average depth of 2.14 ± 0.1 mm^2^ was created [21,38]. This model is used mainly for assessing acute wound healing and cannot be used for the chronic healing process, as the blood vessels remain intact [38]. After inducing the wound, animals were divided into the following groups containing six (06) animals in each group; therefore total 24 rats were used in the experimental study. All 24 rats were used throughout the experimental process. The G*power estimation was used to reach the sample size of six animals per group. Group I represents the negative control (wound-induced rodents) with no treatment; in Group II, the rats were treated with placebo bioactive hydrogel (excluding piperine) twice a day; in Group III, the rats were treated with piperine-containing bioactive hydrogel system (0.5% *w*/*w* of piperine) twice a day; and rats of Group IV were treated with a standard marketed formulation (silver sulfadiazine cream of strength 1% *w*/*w*) twice a day. The percentage of wound contraction was measured on days 1, 5, 9, and 14. Photographs of the wounds were taken for macroscopic observation after each measurement of wound contraction.

Wound Healing % = 1 − (WS_t_/WS_0_) × 100

WS_t_—Wound size on a specific day

WS_0_—Wound size on day 0.

#### 3.5.3. Histopathology and Immunohistochemistry

While performing in vivo studies, the wound size was measured on 1st, 5th, 9th, and 14th day; however, a tissue sample was excised on 14th day only. For excision of sample, the animals were euthanized using diethyl ether inhalation and the sample was taken from the wounded area and analyzed for histopathology. The excised tissues were fixed in 10% buffered formalin for histopathological examination. Subsequently, these tissues were processed by dehydration, wax impregnation, and preparation of blocks with paraffin. The processed tissues were made into sections using a microtome (3–5 µm thick), followed by staining via hematoxylin and eosin. Histological evaluations of the samples were carried out to study the changes induced by various treatments using hematoxylin and eosin stain [22]. The extent of collagen deposition was evaluated in the healed tissued by Masson’s trichrome (MT) Staining. Furthermore, the skin tissue was subjected to immunohistochemical evaluation for Ki-67 expression as marker of cellular proliferation and modulator of wound healing process.

### 3.6. Statistical Analysis

The statistical analysis of study findings was assessed using software (version 6.05, GraphPad Software, Inc., San Diego, CA, USA). The one-way ANOVA followed by Tukey’s multiple comparisons test was applied to demonstrate the statistical significance of the results of different animal groups (*p* < 0.05).

## 4. Conclusions

The present study brings forth the potential role of piperine in the wound healing process. The dispersion of piperine in a hydrogel system containing *Aloe vera* juice further accelerated the wound healing phenomenon significantly. The benefit of the hydrogel as a topical formulation and piperine being a natural bioactive compound ensures the efficacy and safety of the developed formulation system. In vivo and histopathological studies revealed early and intrinsic healing of wounds with the piperine-containing bioactive hydrogel system compared to the bioactive hydrogel system without piperine. The study findings establish the piperine-containing bioactive hydrogel system as a promising therapeutic approach for wound healing applications that needs to be taken to clinical translation for its commercial scalability.

## Figures and Tables

**Table 1:** Percentage Composition of piperine-containing bioactive hydrogel system.

S. No.	Ingredients	Role	% Composition (*w*/*w*)
i.	Carbopol 934	Gelling agent	0.3
ii.	Glycerin	Humectant	5.0
iii.	*Aloe vera* juice	Wound healing promoter	10.0
iv.	Piperine	Bioactive phytochemical	0.5
v.	Triethanolamine	pH adjuster	q.s.
vi.	Distilled water	Vehicle	q.s. to 100

**Table 2:** Percentage (%) of wound contraction in different groups of animals.

Treatment	Day 5	Day 9	Day 14
Group I	4.29 ± 0.62	27.57 ± 0.59	49.98 ± 0.58
Group II	10.17 ± 5.25	40.30 ± 2.20	65.10 ± 1.22
Group III	17.40 ± 0.22	49.58 ± 0.57	80.46 ± 0.87
Group IV	13.43 ± 0.25	49.64 ± 4.27	81.90 ± 2.54

## Data Availability

The data presented in this study are available in the article.
